# Background HIV Incidence in PURPOSE 1 and 2 PrEP Trials

**DOI:** 10.1093/cid/ciaf436

**Published:** 2025-08-14

**Authors:** Jean-Jacques Parienti

**Affiliations:** Department of Biostatistics and Clinical Research, Caen University Hospital, Caen, France; INSERM UMR 1311 DYNAMICURE, Université Caen Normandie, Caen, France

**Keywords:** background HIV incidence, PrEP, counterfactual, design

## Abstract

HIV incidence among low-adherence oral PrEP users in PURPOSE 1 and 2 closely matched or slightly exceeded background HIV incidence (bHIV), supporting bHIV as a conservative comparator in PrEP trials. This enables ethical, efficient evaluation of new prevention strategies without placebo arm.

The design of HIV pre-exposure prophylaxis (PrEP) trials has changed dramatically in recent years. As multiple agents now show near-complete protection when taken sufficiently as prescribed, placebo-controlled trials have become unethical, and noninferiority designs—comparing two active agents head-to-head—often face the practical hurdle of needing impossibly large sample sizes [1].

To address this, a new approach has gained traction with trialists: comparing HIV incidence in the active treatment group to a *counterfactual*—the estimated background HIV incidence (bHIV), also known as cross-sectional incidence that would have occurred without PrEP. bHIV is computed by dividing screening HIV prevalence by the average duration of infection, with adjustments for undiagnosed infections and misclassification [2]. This strategy has been used in PURPOSE 1 [3] and 2 [4], which evaluated lenacapavir and oral PrEP. It is a clever substitute for placebo, but as an indirect comparator approximating the incidence that would be observed in the absence of PrEP, it invites scrutiny.

Our aim was to assess whether the HIV incidence observed with low-level adherence (<2 doses/week)—who were essentially unprotected by PrEP—was consistent with the counterfactual bHIV among PURPOSE [3, 4] participants.

## METHODS

We conducted a post-hoc retrospective analysis based on aggregated data from the oral PrEP arms of the PURPOSE 1 and 2 trials in which adherence was measured via tenofovir diphosphate levels in dried blood spots (DBS): low-level adherence was defined as <2 doses/week (<350 fmol/punch), medium-level as 2–3 doses/week (350–699 fmol/punch), and high-level as ≥4 doses/week (≥700 fmol/punch). The two oral PrEP arms in PURPOSE 1 were combined for better precision.

HIV incidence within adherence strata was estimated using a modeling approach that combined adherence patterns interpolated across time using natural cubic splines, assumed PrEP effectiveness (0% for <2 doses/week, 80% for 2–3 doses/week, 95% for ≥4 doses/week) for previous studies [5–7], and observed trial data. A bootstrap procedure with 1000 iterations accounted for uncertainty in adherence proportions and HIV case counts, simulating HIV infections and computing adherence-stratified incidence rates (per 100 person-years).

We compared the incidence in the lowest adherence group (<2 doses/week) to the background HIV incidence reported in the trial, using normal density distributions based on means and 95% CIs. We computed the overlap coefficient, which ranges from 0 (indicating no overlap) to 1 (indicating identical distributions) representing the shared area under the two density curves. All analyses were performed using R (version 4.5.0).

## RESULTS

**Figure 1.**
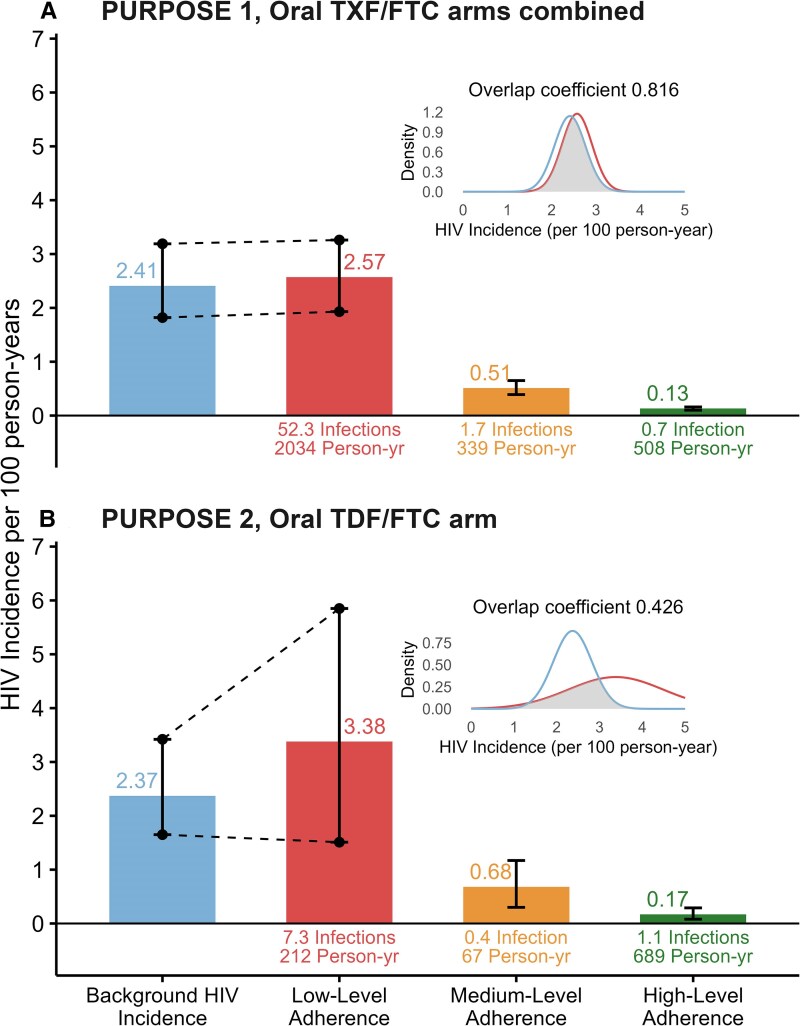
HIV incidence by oral PrEP adherence, PURPOSE 1 (*A*) and PURPOSE 2 (*B*).

As shown in Figure 1, the estimated HIV incidence in the low-adherence group in the PURPOSE 1 oral arms was 2.57 (95% CI: 1.91–3.72) per 100 person-years, compared to a background HIV incidence of 2.41 (95% CI: 1.82–3.19), with an overlap coefficient of 0.82. In the PURPOSE 2 oral arm (Figure 1), the estimated HIV incidence in the low-adherence group was 3.38 (95% CI: 1.51–5.85) per 100 person-years, while the background HIV incidence was 2.37 (95% CI: 1.65–3.42), with an overlap coefficient of 0.43. Across the two trials, point estimates of HIV incidence among low-adherence participants were included within the background HIV incidence 95% CI.

## DISCUSSION

Using published aggregate data from the PURPOSE 1 and 2 trials, we reconstructed a real-world population stratified by adherence to daily oral PrEP. These estimates suggest that background HIV incidence (bHIV) closely mirrors the modeled HIV risk faced by participants with low-level adherence—essentially unprotected—in PURPOSE 1. In contrast, bHIV appears to underestimate HIV risk among low-adherence participants in PURPOSE 2.

This analysis adds to the validity of using bHIV as a placebo-substitute comparator in PrEP trials. Moreover, it reinforces the Bayesian modeling approach used by Glidden et al [7] in the DISCOVER trial, where bHIV was derived from tenofovir diphosphate levels and their established link to HIV protection. Their model also accounted for behavioral confounding, enrollment dynamics, and adherence drift, adding robustness to the comparison.

Interestingly, the overlap coefficient between observed HIV incidence in the low-adherence group and bHIV was much higher in PURPOSE 1 than in PURPOSE 2. This likely reflects key differences between the study populations: cisgender women in PURPOSE 1 versus gay, bisexual men and transgender women in PURPOSE 2. In PURPOSE 1, the low-adherence group comprised a larger fraction of the cohort. For example, 2034 person-year/2881 (70%) of the PURPOSE 1 participants had low-level adherence compared to only 212/968 (22%) in PURPOSE 2 (Figure 1). Therefore, the low-level adherence group was more representative of the overall population in PURPOSE 1 than in PURPOSE 2—and, by extension, the bHIV estimate. In contrast, the low-adherence group in PURPOSE 2 was smaller and the least adherent group may be at the highest background HIV risk potentially inflating incidence in that subgroup. Another possible explanation for this discrepancy is a higher risk compensation behavior following placebo injection, similar to HIV vaccine trials [8] among men compared to women.

That said, a conservative bias in bHIV estimates—underestimating the true risk—may actually be preferable in trial design. If a PrEP intervention outperforms a conservative bHIV estimate, the conclusion holds: the intervention would perform even better against a higher baseline risk.

Limitations include reliance on adherence stratification assumptions, PrEP efficacy estimates, and the uniformity of exposure risk across adherence strata. In addition, the association between adherence-PrEP exposure and efficacy among women has been less studied than among men [9]. However, our analysis of PURPOSE 1 suggests very similar adherence-efficacy relationship. We also lacked participant-level data and relied on summary statistics, limiting precision. bHIV estimates are limited to the enrollment time frame, assuming stable HIV risk throughout the trial. Finally, the precision of the bHIV depends of the accuracy of recency testing including false recency rate and mean duration of recent infection.

In summary, our analysis supports the conservative estimate of the counterfactual comparator in PrEP trials, which has ethical and methodological implications. In settings where adherence to daily oral PrEP is likely to be suboptimal—due to age, stigma, lifestyle, or structural barriers—offering only long-acting PrEP may be both scientifically valid and ethically preferable. While strong laboratory support and focused recruitment strategies are critical to making this approach both feasible and reliable, the use of bHIV as a counterfactual control in PrEP trials enable smaller and faster studies. As prevention strategies expand and the epidemic shifts, using bHIV as a comparator preserves the scientific rigor of a placebo-controlled trial—without exposing participants unlikely to benefit from oral PrEP to unnecessary risk or missed opportunities.
